# Resonant hierarchies: a multiscale framework for oscillatory dynamics in the brain

**DOI:** 10.3389/fpsyg.2026.1704370

**Published:** 2026-01-30

**Authors:** Adam C. Snyder

**Affiliations:** 1 Department of Brain and Cognitive Sciences, University of Rochester, Rochester, NY, United States; 2 Department of Neuroscience, University of Rochester, Rochester, NY, United States; 3 Center for Visual Science, University of Rochester, Rochester, NY, United States

**Keywords:** neural oscillations, dendritic resonance, cortical hierarchy, multiscale dynamics, cross-frequency coupling, neural computation

## Abstract

Oscillatory activity is a hallmark of neural function across spatial and temporal scales, but its origins and computational roles remain only partially understood. Since our earlier caution against treating alpha-band activity as a unitary phenomenon, converging work has highlighted the need to interpret brain rhythms within their anatomical and functional context. Here we provide both a comprehensive review of this progress and a perspective-style framework, *the resonant hierarchy*, which situates oscillations within a nested scaffold spanning from dendritic microstructure to macroscale inter-areal coordination. At the cellular level, dendritic branches act as spatially organized filters with frequency-selective resonance properties. At larger scales, conduction delays and anatomical layout constrain dominant communication frequencies, aligning structural hierarchy with temporal coordination regimes. We argue that canonical rhythms (alpha, beta, gamma, etc.) should be understood not as fixed cognitive modules, but as emergent descriptors of these coordination regimes. In contrast to previous multiscale accounts that focus primarily on laminar microcircuits or network-level eigenmodes, we explicitly link dendritic resonance, laminar organization, and long-range conduction delays into a single cross-scale framework and articulate how they jointly shape latent population dynamics. This perspective unifies diverse findings and generates testable predictions: manipulations of dendritic resonance should systematically shift network oscillations; disruptions of conduction pathways should alter inter-areal alignment; and targeted neuromodulation may work best by nudging latent dynamics along resonant dimensions. In integrating review with framework, we aim to reposition oscillations as fundamental scaffolds of computation, offering a principled basis for future modeling, measurement, and intervention.

## 1 Introduction

Oscillations are a ubiquitous feature of neural activity, observed at scales ranging from subthreshold membrane potential fluctuations to large-scale field potentials recorded from the scalp (Buzsáki and Draguhn, 2004; Foxe and Snyder, 2011; Johnston et al., 2022; Niedermeyer and da Silva, 2005). For decades, neural oscillations have been linked to a variety of cognitive and behavioral processes (Klimesch, 1999; Ward, 2003; Buzsáki, 2006), leading to an interpretive tradition in which specific frequency bands are associated with specific functions. Alpha rhythms, for example, have often been identified with sensory suppression during selective attention (Foxe and Snyder, 2011), gamma with sensory binding (Tallon-Baudry et al., 1999), and theta with episodic memory (Buzsáki, 2002). While such mappings have generated productive hypotheses, they risk oversimplifying the relationship between oscillatory dynamics and neural computation. By tying frequency bands to fixed cognitive roles, this view obscures the possibility that shared temporal constraints, rather than identical computational functions, underlie the recurrence of a given rhythm across diverse contexts.

In our earlier work about the role of alpha oscillations (Foxe and Snyder, 2011), we cautioned that “alpha activity is far from a unitary phenomenon, and discussions of alpha must take into account the anatomical and behavioral context.” Since then, converging evidence has reinforced the idea that oscillations should be interpreted within their anatomical, physiological, and behavioral context (Snyder et al., 2018; Bastos et al., 2015; Buzsáki, 2010; Samaha and Romei, 2024). Building on this foundation, we propose here a more general framework: *resonant hierarchies*. Rather than assigning each frequency band a fixed computational role, this framework situates oscillations within a nested set of structural and dynamical constraints spanning dendritic compartments, microcircuits, and distributed brain networks. Within this view, a given rhythm's most fundamental descriptor is the temporal coordination regime it instantiates, which may serve multiple functions to the extent they share similar timing demands.

The resonant hierarchy framework is motivated by two complementary developments. First, advances in multiscale recording have revealed systematic relationships between spatial scale, anatomical structure, and dominant oscillatory frequencies (Johnston et al., 2022; Spaak et al., 2012; Buffalo et al., 2011). Second, theoretical and computational work ranging from laminar-specific communication models to analyses of conduction delays has begun to link these relationships to the flow of information in the brain (Snyder et al., 2018; Roberts et al., 2013; Buzsáki and Draguhn, 2004). These insights resonate (pardon the pun) with Buzsáki's (2006) call to “reverse-engineer” the brain by beginning with its physical constraints, rather than seeking neural correlates of predefined cognitive modules. By aligning oscillatory coordination with the anatomical and biophysical hierarchies of the nervous system, we suggest it is possible to recover a set of computational primitives that cut across conventional functional categories.

The present article is best read as a *perspective* that integrates these strands of evidence into a common organizing framework. Our primary goal is to synthesize existing multiscale work on oscillations, ranging from laminar-specific models to whole-brain connectivity analyses, into the language of resonant hierarchies that explicitly connects dendritic resonance, mesoscale circuitry, and long-range communication. In doing so, we aim to provide a conceptual bridge between cellular and systems-level accounts of brain rhythms that is accessible to cognitive scientists while remaining grounded in biophysics and anatomy.

In the sections that follow, we first review the large-scale principles governing inter-areal communication, emphasizing how anatomical layout, conduction delays, and cortical hierarchy jointly constrain the dominant temporal coordination regimes between areas. We then turn to the microscale, examining how gradients in dendritic channel density, morphology, and active conductances create compartment-specific resonance profiles, and how these properties interact with local microcircuit computations. Next, we integrate these perspectives into a multiscale resonant hierarchy framework, highlighting reciprocal interactions and plasticity mechanisms that couple dynamics across levels and clarifying how this framework extends prior multiscale models of oscillations. We close by considering experimental and modeling approaches for probing these links, articulating testable predictions, and exploring potential clinical and translational implications. By grounding oscillatory phenomena in scale-dependent resonant properties, we aim to provide a principled account of why particular rhythms emerge in specific anatomical and functional contexts, and how these rhythms might be leveraged in future interventions.

## 2 Hierarchical brain rhythms and their functional roles

### 2.1 Canonical oscillatory bands and associated functions

Oscillatory activity in the brain spans a broad range of frequencies, and certain bands recur across species, brain regions, and behavioral contexts (da Silva, 1991; Buzsáki and Draguhn, 2004; Siegel et al., 2012). While their precise boundaries vary somewhat between studies, the most widely recognized bands (delta, theta, alpha, beta, and gamma) are associated with distinct, though sometimes overlapping, functional roles.

**Delta** rhythms (~0.5–4 Hz) dominate during deep non-REM sleep and low-arousal states (Steriade et al., 1993; Olbrich et al., 2003). They are thought to provide a global temporal framework for neural activity, coordinating widespread cortical and subcortical structures over long timescales (Buzsáki and Draguhn, 2004; Lakatos et al., 2005). In waking states, delta oscillations can emerge during tasks requiring sustained attention under low arousal, potentially reflecting large-scale integrative processes that operate on slow temporal windows (Harmony, 2013).

**Theta** rhythms (~4–8 Hz) are prominent in hippocampal and fronto-parietal networks, where they have been linked to attentional orienting, spatial navigation, and the temporal organization of episodic memory (Buzsáki, 2005; Kahana et al., 1999; Fiebelkorn et al., 2013; Fiebelkorn and Kastner, 2019). Theta oscillations are also implicated in switching the locus of attention or intention, possibly by segmenting processing into discrete temporal epochs (Sauseng et al., 2010; Caplan et al., 2003). Recent work has highlighted “theta sweeps” as a potential mechanism for prospective decision-making and future planning in hippocampo-cortical circuits, in which sequential representations of candidate trajectories unfold within each theta cycle (Kay et al., 2020; Vollan et al., 2025). More broadly, theta may support flexible shifts between external and internal attentional states (Nobre and Gresch, 2025), and in working memory contexts, theta plays an active role in anticipatory control across spatial scales (Jung et al., 2025).

**Alpha** rhythms (~8–13 Hz) are especially prominent in occipital and parietal cortices during wakeful rest, but their amplitude is modulated by attention and task demands. A substantial body of evidence supports the view that alpha-band activity functions as a sensory suppression or gating mechanism, selectively inhibiting task-irrelevant processing while facilitating relevant inputs (Foxe and Snyder, 2011; Banerjee et al., 2011; Snyder and Foxe, 2010; Snyder et al., 2015; Klimesch, 2012). Alpha is also frequently observed in cross-frequency coupling with faster oscillations, particularly gamma, which may enable temporal framing of local computations by large-scale inhibitory rhythms (Fiebelkorn et al., 2018; Spaak et al., 2012; Roux et al., 2013; Bastos et al., 2018).

**Beta** rhythms (~13–30 Hz) are often observed in sensorimotor and fronto-parietal networks. They have been linked to the maintenance of the current sensorimotor or cognitive set, and the integration of information across modalities (Engel and Fries, 2010; Spitzer and Haegens, 2017; Haegens et al., 2017). Beta activity often decreases prior to movement initiation and rebounds afterward, suggesting a role in both maintaining and resetting network states (Pfurtscheller et al., 1996; Salmelin et al., 1995; Kilavik et al., 2013). Recent work has shown that rhythmic temporal coordination in the beta band can prevent representational conflict during working memory by allocating competing neural representations to distinct phases of the cycle (Abdalaziz et al., 2023; Bastos et al., 2018), and may also support top-down control and cross-scale coordination (Jung et al., 2025).

**Gamma** rhythms (≳30 Hz) are typically localized to relatively small cortical domains and have been associated with feedforward processing, fine-scale sensory encoding, and the precise temporal coordination of spiking activity. Early work in visual cortex suggested a role in feature binding (Tallon-Baudry et al., 1999; Singer, 1999), while other studies have emphasized gamma's role in enhancing effective connectivity and communication between neuronal groups (Fries, 2005). Gamma-band synchronization has been observed in both sensory and association areas during attention-demanding tasks (Bosman et al., 2012; Gregoriou et al., 2009), and its laminar organization supports preferential involvement in feedforward pathways (Bastos et al., 2015; Spaak et al., 2012). Recent work has examined how gamma interacts with slower rhythms, such as theta or alpha, to embed high-resolution computations within broader temporal frameworks (Colgin et al., 2009; Fiebelkorn et al., 2018).

These associations are not exclusive, and each frequency band can participate in multiple functions. However, their recurring association with specific aspects of perception, cognition, and action provides a functional scaffold for understanding oscillatory hierarchies. Although these associations provide a useful scaffold for interpreting canonical bands, it is important to note that some remain debated, and there are studies and reviews that challenge or complicate these functional assignments (e.g., Ray and Maunsell, 2010; Merker, 2013). In the framework developed here, these canonical bands are not arbitrary divisions of the spectrum, nor fixed signatures of particular functions, but rather reflect constraints imposed by structural and dynamical properties of the nervous system across multiple spatial scales.

### 2.2 Nested and interdependent rhythms

Oscillations in different frequency bands do not operate in isolation. Instead, they interact in structured ways that enable coordination across spatial and temporal scales. One of the most widely studied forms of interaction is *cross-frequency coupling*, in which the phase or amplitude of one oscillation systematically covaries with the phase or amplitude of another at a different frequency (Jensen and Colgin, 2007; Canolty and Knight, 2010; Riddle et al., 2021). Classic examples include theta–gamma coupling in the hippocampus (Bragin et al., 1995; Colgin et al., 2009) and alpha–gamma coupling in visual cortex (Osipova et al., 2008; Spaak et al., 2012). In these cases, slower rhythms appear to organize the timing or magnitude of faster activity, creating a temporal framework within which fine-scale computations unfold.

Two common forms of such coupling are *phase–amplitude coupling*, where the amplitude of a faster oscillation is modulated by the phase of a slower one, and *phase–phase coupling*, where the relative phase relationship between two oscillations remains consistent over time (Canolty and Knight, 2010; Jensen and Colgin, 2007). These mechanisms can facilitate selective information routing, as the temporal windows in which a fast rhythm is strongest can be aligned with specific phases of a slower rhythm that correspond to heightened excitability or communication readiness (Fiebelkorn and Kastner, 2019; Roux et al., 2013; Lakatos et al., 2005; Axmacher et al., 2010; Bastos et al., 2018).

**Figure 1:**
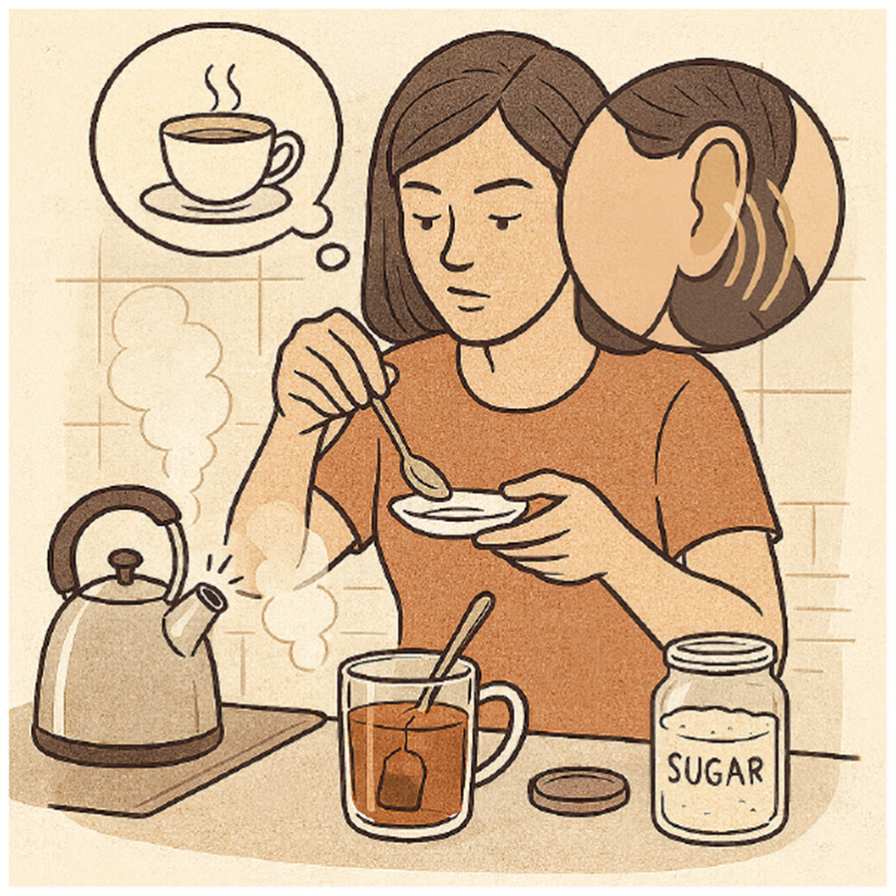
Computations supporting cognition are intrinsically hierarchical. Maintaining a task set (“make tea”) guides working memory (keeping track of the sequence, “boil water, steep tea, add sugar”), which prioritizes sensory processing (listening for the kettle whistle).

The dependencies among rhythms often form a hierarchy, with the slowest oscillations exerting broad, global influences and faster rhythms providing local, high-resolution processing. This nested structure of oscillations may reflect more than just a biophysical constraint; it may represent a natural alignment between the temporal scales of neural dynamics and the hierarchical structure of brain computations. At the computational level as described by Marr and Poggio (1977), many brain functions are themselves hierarchically organized: higher-level goals or contexts set the conditions for intermediate operations, which in turn guide more elemental processes. For example, maintaining a task set can constrain the sequencing of working memory operations, which in turn modulates moment-to-moment sensory selection (Figure 1). Such hierarchies of function require coordination across multiple timescales, and the brain's oscillatory hierarchy may be specifically tuned to meet this demand. From this perspective, the alignment of slower, large-scale rhythms with long-horizon computations, and faster, local rhythms with fine-grained processing, is not coincidental but a natural outcome of a system whose physical and computational architectures are both intrinsically hierarchical.

## 3 Spatial embedding and communication constraints

### 3.1 Conduction delay and frequency matching

The spatial arrangement of neural circuitry imposes fundamental constraints on the timing of communication between brain regions. Axonal conduction velocities vary widely depending on fiber diameter and myelination (Waxman, 1980), but even under optimal conditions, transmission over long distances incurs measurable delays (Swadlow, 2000). For example, signals traveling between distant cortical areas may require several milliseconds to complete a round trip (Caminiti et al., 2009; Budd and Kisvárday, 2012), whereas local circuit interactions can occur within fractions of a millisecond (Feldmeyer et al., 1999).

**Figure 2:**
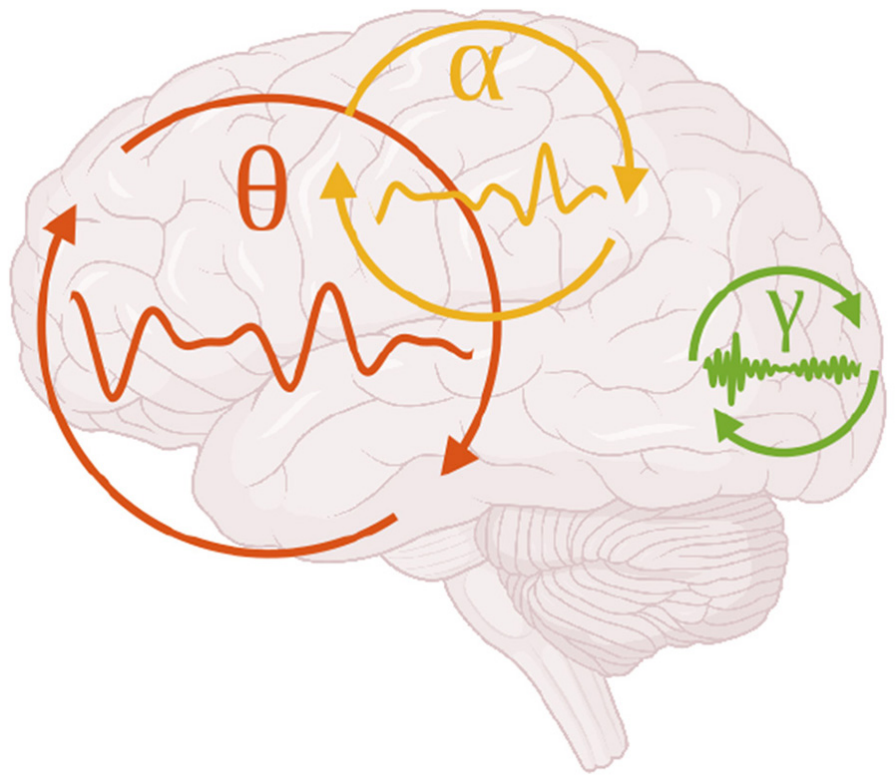
The oscillatory frequency of interaction between brain areas tends to decrease as the distance between brain areas increases. For example, coupling between V1 and V2 in the gamma band (Roberts et al., 2013, green), fronto-parietal coupling in the alpha band (Palva and Palva, 2011, yellow), and hippocampal-frontal coupling in the theta band (Jones and Wilson, 2005, red).

These delays place limits on the range of oscillation frequencies that can effectively coordinate activity between regions (Petkoski and Jirsa, 2019). High-frequency rhythms, such as gamma, have short cycle durations and are therefore most compatible with interactions among nearby neurons and local cortical circuits, where conduction delays are negligible relative to the oscillation period. In contrast, long-range interactions such as those linking frontal and parietal cortices require slower rhythms to maintain consistent phase relationships, making them better suited to beta, alpha, or even theta frequency ranges (Figure 2).

This relationship between conduction distance and optimal communication frequency has been supported by computational models and empirical observations. Studies have shown that coherence between distant cortical sites is more pronounced at lower frequencies, whereas local field potentials within a region or across adjacent areas exhibit stronger high-frequency synchronization (Roberts et al., 2013; Buzsáki and Draguhn, 2004; Myers et al., 2022). In this way, anatomical distance acts as a natural filter on the frequency channels available for inter-areal coordination, embedding spatial constraints directly into the temporal architecture of large-scale brain rhythms.

An important refinement of the distance–frequency relationship comes from studies examining the directionality of inter-areal communication. Feedforward interactions (from areas “lower” in the visual processing hierarchy to areas “higher” in that hierarchy) are preferentially mediated by gamma-band coherence, whereas feedback interactions from higher to lower areas are mediated by alpha- or beta-band coherence (Bastos et al., 2015; Michalareas et al., 2016). Causal manipulations provide converging evidence: in laminar electrode array recordings from visual areas V1 and V4, van Kerkoerle et al. (2014) demonstrated that intracortical electrical microstimulation delivered in the gamma band preferentially propagated in the feedforward direction, while alpha- or beta-frequency stimulation preferentially propagated in the feedback direction. These findings suggest that cortical networks exhibit direction-selective frequency tuning, such that the circuitry involved in feedforward or feedback communication resonates more effectively to input at the frequencies it normally carries.

This direction–frequency mapping is closely linked to the laminar microcircuitry of the cortex. Functionally, information flow between brain areas in the feedforward and feedback directions relies on different mixtures of neurons within each brain area (Semedo et al., 2022). Anatomically, feedforward projections typically originate in superficial layers and target layer 4 of recipient areas, while feedback projections arise from deep layers and terminate in superficial layer 1 and deep layer 6 (Felleman and Van Essen, 1991; Markov et al., 2013). Cellular and synaptic properties of neurons in these layers may predispose them to preferentially engage in distinct frequency bands, thereby creating layer-specific frequency channels for inter-areal communication (more on this below). Functionally, laminar differences in oscillatory coherence and spike–field coupling further support this division of labor across frequency and scale (Senzai et al., 2019). In this way, the feedforward/feedback dissociation in oscillatory frequency can be viewed as a mesoscale instantiation of the broader resonant hierarchy principle, where structural and biophysical properties at the cellular and laminar levels shape the temporal modes of large-scale network interactions.

### 3.2 Structural hierarchy of inter-areal communication

The arrangement of cortical and subcortical areas into a structural hierarchy is often discussed in terms of wiring efficiency and developmental constraints (Cherniak et al., 2004; Budd and Kisvárday, 2012; Chen et al., 2006; Samu et al., 2014), but this architecture may also serve to align each subsystem with an appropriate temporal regime for its computational role. Functionally specialized regions are embedded in the cortex at varying anatomical distances from one another, positioning them to interact preferentially at frequencies suited to the demands of their computations.

Sensory hierarchies provide a clear example. Adjacent visual areas such as V1 and V2, which exchange large volumes of information about fine-scale spatial features, are connected by short, direct pathways and often synchronize in the gamma band (Roberts et al., 2013). These high-frequency interactions support rapid, temporally precise encoding and feedforward integration. By contrast, association networks such as the fronto-parietal loop operate over longer conduction distances and are more frequently linked by beta- or alpha-band coordination, consistent with their role in maintaining and updating task sets over hundreds of milliseconds (Jung et al., 2025; Palva and Palva, 2011; Spitzer and Haegens, 2017; Sehatpour et al., 2008; Phillips et al., 2014). At the largest scale, hippocampal–prefrontal pathways implicated in episodic memory and prospective planning coordinate over theta (and to some extent delta) rhythms, enabling the integration of information streams across time periods on the order of seconds (Jones and Wilson, 2005; Colgin, 2011; Fujisawa and Buzsáki, 2011; Tang et al., 2021; O'Neill et al., 2013; Domanski et al., 2023).

Work on large-scale functional networks and structural connectomes provides converging support for these ideas. Resting-state fMRI and electroencephalography (EEG)/magnetoencephalography (MEG) studies have shown that functional connectivity patterns can be understood, in part, as arising from oscillatory dynamics unfolding on a fixed anatomical scaffold, with conduction delays and network topology shaping the emergence of band-limited networks (e.g., Deco et al., 2011; Cabral et al., 2017; Tewarie et al., 2019). Recent analyses have further highlighted the role of structural eigenmodes in organizing these networks, suggesting that distinct frequency-specific networks may correspond to different eigenmodes of the same underlying structural connectome (Cordier et al., 2024). Although fMRI lacks the temporal resolution to resolve individual oscillatory cycles, its resting-state literature has been essential in showing that structural connectivity and conduction-delay constraints shape large-scale network modes, providing a complementary view to electrophysiological studies. From the resonant hierarchy perspective, these results underscore how macroscale constraints on oscillatory modes interact with local dendritic and microcircuit mechanisms, jointly determining which temporal coordination regimes are expressed at rest and during task performance.

The correspondence between anatomical position, frequency of interaction, and computational role suggests that the spatial embedding of functional subsystems reflects an optimization in which the physical placement of areas in the cortical hierarchy naturally constrains them to operate within temporal coordination regimes that match their functional specializations. Such scale-specific constraints need not apply only at the level of whole-brain networks. As we turn to the next level of analysis, similar principles emerge within the morphology and conductances of individual neurons. Here, frequency tuning arises not from inter-areal conduction delays, but from the resonant properties of dendrites and somata themselves, providing a local frequency scaffold that can align with, and be modulated by, the larger-scale oscillatory architecture.

## 4 Dendritic resonance as a frequency scaffold

### 4.1 Biophysical basis of dendritic resonance

Resonance in neuronal membranes arises from the interplay of passive cable properties and active voltage-dependent conductances that confer frequency-selective responsiveness (Hutcheon and Yarom, 2000; Hutcheon et al., 1996; Hu et al., 2002). Among the most studied contributors to subthreshold resonance are the hyperpolarization-activated cation current (*I*_*h*_; Pape, 1996; Magee and Johnston, 1995), the muscarine-sensitive potassium current (*I*_*M*_; Halliwell and Adams, 1982; Yue and Yaari, 2004; Vera et al., 2017), and low-threshold T-type calcium currents (*I*_*CaT*_; Hutcheon et al., 1994; Perez-Reyes, 2003; Matsumoto-Makidono et al., 2016). These conductances introduce nonlinearity and temporal filtering into the membrane response, allowing cell membranes to exhibit a peak resonance at specific input frequencies depending on the cell type and compartment (Remme et al., 2009). For example, *I*_*h*_ currents, which activate upon hyperpolarization and carry a mixed Na^+^/K^+^ inward current, can counteract low-frequency membrane potential deflections, effectively acting as a high-pass filter (Pape, 1996; Magee and Johnston, 1995). When combined with membrane capacitance and leak conductances, such currents yield a band-pass filtering effect that preferentially transmits (or even amplifies) inputs around a preferred frequency while attenuating others (Hutcheon and Yarom, 2000; Hutcheon et al., 1996).

Importantly, these resonance properties are not fixed. They can be dynamically modulated by neuromodulators such as dopamine, which shifts *I*_*h*_ activation (Rosenkranz and Johnston, 2006), and acetylcholine, which suppresses *I*_*M*_ (Delmas and Brown, 2005), as well as by activity-dependent plasticity. For instance, resonance frequency in hippocampal pyramidal neurons can be bidirectionally tuned by experience through long-term regulation of *I*_*h*_ conductances (Narayanan and Johnston, 2007, 2010). Thus, resonance is not simply a passive feature of neural membranes but a tunable property that may serve context-dependent computational roles. In the context of the resonant hierarchy framework we propose here, such intrinsic frequency tuning at the level of dendrites and somata provides a substrate upon which larger-scale oscillatory coordination may be scaffolded.

### 4.2 Spatial dependence of resonant properties

**Figure 3:**
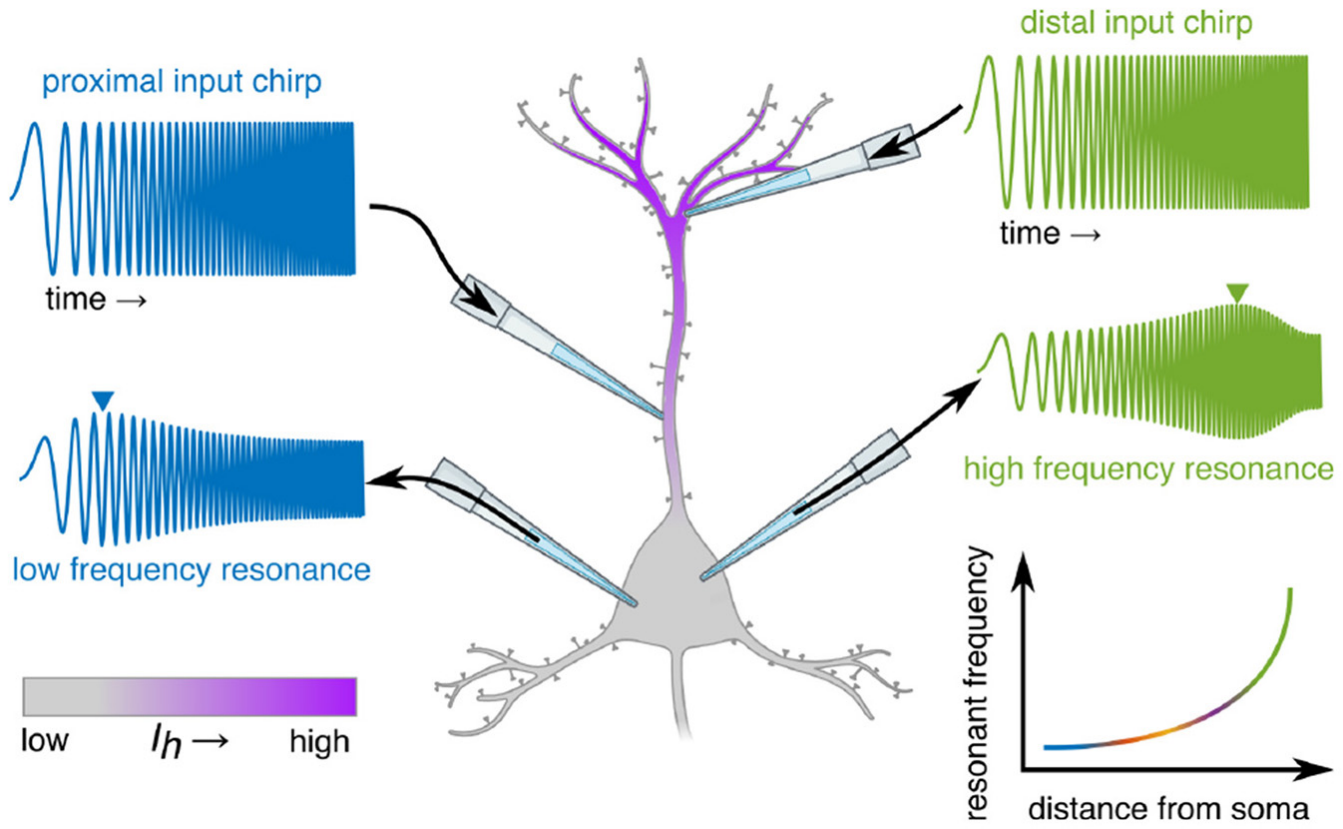
Conceptual illustration of measuring dendritic resonance. Dendritic resonance is measured by inputting a linear frequency “chirp” at varying positions along the dendrite, and measuring the resultant voltage at an integration zone, such as the soma (e.g., Narayanan and Johnston, 2007), which is attenuated at some frequencies and preserved/amplified at others (the “resonant frequency,” inverted triangles). This resonant frequency tends to increase exponentially with distance from the soma, as does the conductance of *I*_*h*_ (purple gradient).

In many pyramidal neurons, including CA1 hippocampal cells, the density of hyperpolarization-activated cation channels (*I*_*h*_) increases with distance from the soma (e.g., Magee, 1998; Narayanan and Johnston, 2007). Because *I*_*h*_ activation confers a preference for faster membrane potential fluctuations (Hu et al., 2009), distal compartments tend to resonate at higher frequencies than proximal compartments or the somata (Narayanan and Johnston, 2007) (Figure 3). This pattern arises from the interaction between local input impedance, passive cable filtering, and the spatial distribution of active conductances.

Even in the simplest case of a uniform resonant cable, spatial separation between an input site and the soma produces a measurable shift in the preferred frequency of the transfer impedance, on the order of ten percent over a few hundred micrometers as revealed by computational models by Laudanski et al. (2014). Those researchers emphasized that this passive gradient is only a lower bound. When realistic dendritic features are introduced in the models, including branching, nonuniform distributions of resonant conductances, and somatic boundary conditions, the resonance profile varies much more sharply, often by tens of percent across individual branches in both abstract and reconstructed morphologies. In those models, resonant frequencies often occurred in very high ranges (for example, above 200 Hz), but the key point is less about the absolute values in the simplified models than the broader principle that multiple biophysical and geometrical factors contribute to spatial dependence of dendritic resonance. Importantly, recordings from biological neurons reveal gradients that fall within canonical physiological ranges. Narayanan and Johnston (2007), for example, reported a shift from roughly 2 Hz at the soma to roughly 12 Hz at distal apical dendrites within approximately 300 micrometers. Such variation creates a spatially structured temporal filter that can differentially emphasize distinct components of synaptic input. Synaptic inputs arriving on distal dendrites are thus more likely to be amplified when their temporal fluctuations match the local higher-frequency resonance, whereas lower-frequency components are attenuated. Conversely, inputs impinging on proximal dendrites or the soma align better with the lower-frequency resonance found in those regions. This gradient means that inputs targeting different compartments will preferentially engage different temporal modes.

Anatomically, the relationship between projection distance and dendritic targeting is more nuanced than previously assumed. Individual corticocortical axons often arborize across multiple layers, engaging distinct dendritic compartments of their postsynaptic targets (Rockland and Virga, 1989). Because dendritic compartments exhibit frequency-selective resonance properties, a single presynaptic neuron could differentially influence them depending on its firing frequency. In effect, arborization combined with dendritic filtering allows one axon to multiplex its output across parallel computational channels within dendrites. It should also be noted that axonal arbors are not merely passive “splitters” of presynaptic activity but hierarchical structures capable of their own computations, though a full treatment of this complexity is beyond the present focus.

Within the apical arbors of layer 3 pyramidal neurons, long-range excitatory inputs have been shown to cluster in discrete domains arranged systematically along the apical axis, with more centrally originating axons innervating progressively more distal dendritic regions (Petreanu et al., 2009). These findings suggest that long-range connections may differentially recruit proximal versus distal dendritic compartments, each of which exhibits distinct resonance properties. At the macro-scale, long-range interactions are typically dominated by lower-frequency rhythms, consistent with conduction delays and the integrative demands of distributed processing (see above). In this way, dendritic resonance and axonal arborization patterns jointly support multiplexed computation, bridging micro-scale dendritic dynamics with the macro-scale hierarchy of cortical oscillations.

However, dendritic compartments are not passive relays for single-source input (e.g., either local or long-range). Instead, they can perform frequency-selective integration across convergent streams, with harmonic relationships and cross-frequency nesting enabling slower rhythms to modulate the excitability of locally driven fast activity. Consistent with this idea, distal apical tufts in layer 1 are positioned to integrate both local high-frequency inputs and slower long-range feedback, supporting their role as convergence sites for distributed computations (Cauller, 1995).

### 4.3 Computational implications of dendritic structure

The dendritic arbor is an active computational substrate whose structure and conductances shape the neuron's information processing capabilities. Local mechanisms such as shunting inhibition can selectively attenuate or veto the impact of nearby inputs. Early theoretical work proposed that this synaptic veto could underlie selective computations such as orientation tuning in cortex (Koch and Poggio, 1985). Empirically, intracellular recordings in visual cortex confirmed that visual input evokes strong, transient shunting inhibition, directly demonstrating its capacity to sculpt local input integration (Borg-Graham et al., 1998). More recent modeling and experimental analyses have shown how dendritic geometry and inhibitory placement govern the efficacy of shunting, clarifying the principles by which such inhibition shapes synaptic integration in dendrites (Gidon and Segev, 2012). By increasing local membrane conductance, these inhibitory synapses reduce input resistance and shorten the electrotonic space constant, limiting the spread of voltage changes and thereby modulating the influence of other inputs within the same branch. Active processes such as N-methyl-D-aspartate (NMDA) spikes (Schiller et al., 2000), calcium spikes (Larkum et al., 1999; Gidon et al., 2020), backpropagating action potentials (Stuart et al., 1997), and other forms of dendritic electrogenesis (reviewed in London and Häusser, 2005) can locally amplify coincident inputs, creating branch-specific nonlinearities that function as conditional gates or coincidence detectors.

The hierarchical branching of dendrites is reflected in the etymology of *dendrite*, derived from the Greek *dendron*, meaning “tree,” which is arguably the quintessential hierarchical structure. This branching pattern naturally supports a layered form of integration. Sub-branches can process inputs semi-independently, performing local computations before passing their results toward higher-order branch points (e.g., Mel, 1994; Poirazi and Mel, 2001; Polsky et al., 2004; Losonczy and Magee, 2006). These intermediate nodes then integrate information from multiple sub-branches before relaying it toward the soma. This structural hierarchy enables the neuron to implement multi-stage transformations: distal branches may act as feature detectors for specific input patterns, while more proximal regions integrate these features in the context of broader synaptic activity.

**Figure 4:**
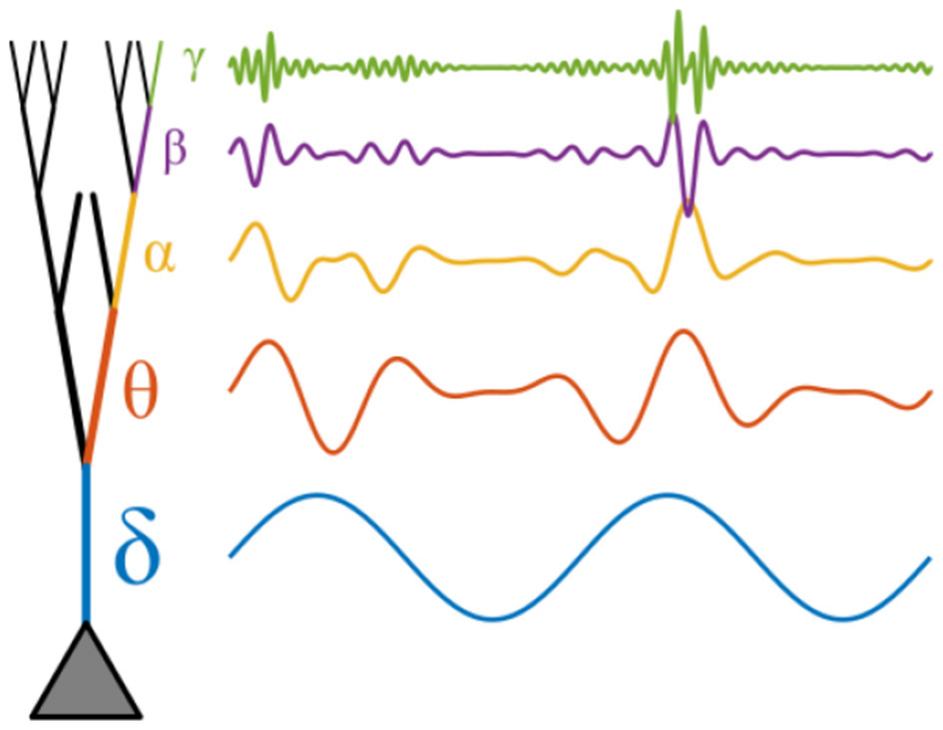
Conceptual illustration of potential phase-amplitude coupling mechanism in an idealized hierarchical dendrite. Inputs are filtered based on the resonance phenomena at different dendritic compartments. The effect of input at more distal locations (higher frequencies) is modulated by the phase of input at more proximal compartments (lower frequencies), due to mechanisms such as shunting inhibition.

Because local computations are shaped by the branch's resonant properties, different parts of the dendritic tree may be tuned to detect and integrate inputs with distinct temporal signatures, providing a natural division of labor across frequencies. Harmonic relationships between these locally preferred frequencies may further enable coordinated computation across dendritic compartments. For example, a proximal branch resonating in the theta range could modulate the gain or timing of inputs to a distal branch tuned to gamma frequencies, effectively nesting fast feature detection within a slower integrative cycle (Figure 4). Such cross-frequency coupling can allow temporally distinct processes (e.g., local coincidence detection and broader contextual integration) to remain phase-aligned, preserving the computational benefits of both. In this way, the dendritic tree can support multiplexed operations in which nested oscillatory interactions coordinate diverse computations across spatially and biophysically distinct compartments.

These capabilities parallel the principles of hierarchical cortical processing: local, frequency-specific processing at the periphery feeding into broader, integrative decision nodes nearer the soma. In this view, the neuron is not a single summing point but a structured network in its own right, capable of context-dependent gating, temporal filtering, and selective amplification. Each of these processes can interact with and contribute to the organization of network-level oscillations.

## 5 Resonant hierarchies: a multiscale synthesis

### 5.1 Alignment across scales

A central premise of the resonant hierarchy framework is that oscillatory coordination is organized along parallel structural hierarchies spanning multiple spatial scales (summarized in Table 1). Related multiscale proposals have emphasized how resonance can arise and interact across levels, from intrinsic membrane currents to recurrent microcircuits and structured long-range networks (e.g., Stark et al., 2013, 2022). Stark and colleagues provide a detailed mechanistic account of how resonant motifs may be generated, inherited, or independently instantiated at different scales, and how these motifs contribute to band-limited interactions within cortical hierarchies. Here we adopt a complementary perspective. Rather than focusing on the mechanistic routes by which resonance emerges within particular circuits, we treat dendritic resonance, laminar architecture, long-range conduction delays, and latent population dynamics as parallel tiers within a unified scaffold, and ask how constraints at these levels jointly align the temporal coordination regimes that organize computation across scales. This difference in emphasis means that the resonant hierarchy framework is less a model of a particular circuit and more a cross-scale organizing principle intended to guide both mechanistic modeling and experimental design.

**Table 1:** Summary of conceptual parallels of oscillatory resonance across scales spanning subcellular computation in dendrites, to large-scale networks, to computational functions.

**Frequency band**	**Function(s)**	**Dendrites**	**Large-scale networks**
δ (1–4 Hz)	Arousal/slow integration; sleep homeostasis	Proximal/somatic integration; long windows	Global coordination; high delay tolerance
θ (4–8 Hz)	Sequencing; navigation; working memory control	Mid-apical coupling; θ–γ nesting	Hippocampal–PFC loops; cross-areal timing
α (8–14 Hz)	Gating/selection; sensory suppression	Compartment-specific resonance; gain control	Feedback-dominant channels; mesoscale coherence
β (15–30 Hz)	Set-maintenance; pre-movement decrease; rebound	Dendrite–soma coupling for top-down “set”	Long-range control; fronto-parietal loops
γ (>30 Hz)	Feature binding; local computation; assemblies	Distal/apical tuft engagement; fast resonance	Feedforward precision; short-range routing

At the microscale, dendritic resonance properties impose frequency preferences on synaptic inputs, shaping which temporal patterns are most likely to influence somatic integration. At the mesoscale, local columns and laminar circuits exhibit intermediate-scale resonances, arising from recurrent connectivity and layer-specific conductances. At the macroscale, inter-areal coordination is constrained by conduction delays, axonal pathways, and anatomical layout, producing systematic relationships between spatial separation and dominant communication frequencies. This multi-scale view reframes the interpretation of canonical frequency bands: rather than assigning alpha (or any other oscillatory band) a fixed role derived from a particular behavioral paradigm, we propose that an oscillation's most fundamental descriptor is the temporal coordination regime it embodies. In this view, a given frequency band may participate in diverse functions, provided those functions share common temporal constraints. This shift echoes a broader conceptual reorientation articulated by Buzsáki (2006), who noted that neuroscience inherited from psychology a set of predefined cognitive “modules” (e.g., attention, working memory) and sought neural correlates for each. An alternative, he suggested, is to begin with the brain as a physical system (with its anatomical layout, conduction delays, and resonant properties) and reverse-engineer the computational primitives it naturally supports. The resonant hierarchy framework follows this latter approach, rooting large-scale functional diversity in a shared set of scale-dependent dynamical motifs, rather than mapping rhythms one-to-one onto high-level cognitive constructs.

This alignment across scales suggests that the brain's oscillatory architecture is not an incidental byproduct of neural activity but an evolved, multi-level organizational principle, reflecting constraints imposed by both neural biophysics and the intrinsic structure of computation itself. Frequency preferences originating at the level of dendritic compartments can propagate upward, influencing the collective dynamics of microcircuits, cortical areas, and whole-brain networks. Conversely, the global oscillatory environment can modulate, entrain, or adapt the resonance properties of its cellular and subcellular components.

### 5.2 Reciprocal interactions and plasticity

Oscillatory dynamics are not only shaped by the structural and resonant properties of their neural substrates; they also act back upon those substrates to modify their future responsiveness. Top-down rhythms can modulate dendritic integration through multiple mechanisms, including activity-dependent synaptic plasticity (Huerta and Lisman, 1995; Buzsáki and Draguhn, 2004), changes in intrinsic excitability via conductances such as *I*_*h*_ and *I*_*M*_ (Hutcheon and Yarom, 2000; Narayanan and Johnston, 2010), neuromodulator-driven shifts in channel properties (Marder, 2012; Dembrow et al., 2010), and even ephaptic coupling between neighboring elements (Anastassiou et al., 2011; Fröhlich and McCormick, 2010).

For example, prolonged entrainment of a dendritic compartment at a particular frequency can upregulate or downregulate ion channel densities, altering its resonance profile and thereby biasing future sensitivity toward the entrained frequency band (e.g., Fan et al., 2005; Remy and Spruston, 2007; Narayanan and Johnston, 2010, 2007; Zhang and Linden, 2003). Similarly, neuromodulatory inputs such as cholinergic or noradrenergic projections can rapidly change time constants and gain control parameters, transiently reshaping resonance without requiring structural change (e.g., Robinson and Siegelbaum, 2003; Biel et al., 2009).

These bidirectional influences likely create a feedback loop in which macroscopic rhythms both emerge from and reshape their generating microcircuits. When large-scale network activity exhibits consistent temporal patterns—such as sustained theta in hippocampal-prefrontal loops (e.g., Colgin, 2011) or alpha in visual-parietal networks (Foxe and Snyder, 2011)—these patterns can leave enduring “traces” in the form of synaptic weight adjustments, changes in dendritic ion channel expression, and reorganization of local microcircuit connectivity. Over time, these traces alter the baseline resonance properties of the contributing neurons, increasing the likelihood that the same large-scale pattern will reoccur. This dynamic is akin to stigmergy in collective systems, where actions taken by the group leave modifications in the environment that bias future behavior in a self-reinforcing manner (Bonabeau et al., 1999).

In the resonant hierarchy framework, such stigmergic loops provide a mechanism for the co-adaptation of oscillatory dynamics and structural architecture across scales. Local dendritic tuning shapes the emergence of mesoscale and macroscale rhythms, while those rhythms, in turn, serve as a sculpting force, reinforcing structural motifs and conductance profiles that support their propagation. This reciprocal interplay reflects a system in which temporal coordination regimes and anatomical specializations are co-constructed over developmental, learning, and evolutionary timescales.

### 5.3 Dimensionality, sampling, and access to global state

Large-scale neural population activity often evolves on a manifold whose intrinsic dimensionality is far lower than the number of constituent neurons (e.g., Gao and Ganguli, 2015; Stringer et al., 2019). If this low-dimensionality assumption holds, then results such as the Johnson–Lindenstrauss lemma (Johnson and Lindenstrauss, 1984; Vempala, 2005; Box 1) imply that a relatively small number of projections into the activity space can preserve the essential geometry of the global state. While dendritic integration is neither perfectly linear nor composed of truly random projections, each neuron receives thousands of inputs from a diverse set of presynaptic partners, spanning both local and long-range sources. This diversity, combined with the filtering properties of dendrites, makes it plausible that the membrane potential of a single neuron can embed a substantial amount of information about the overall network state, even if each neuron accesses only a particular, biased projection of that state. In the context of resonant hierarchies, this observation has important implications. Because dendritic resonance varies spatially along the arbor, different compartments preferentially transmit inputs within distinct frequency bands and from distinct mixtures of presynaptic partners. Large-scale oscillations, in turn, often carry functionally distinct streams of information in different bands, such as gamma for feedforward sensory signals and beta for top-down control. A neuron's structured frequency filtering can therefore act as a targeted sampler of the global latent state, selectively amplifying inputs that are temporally aligned with its local resonances and connectivity profile. Proximal and distal branches, and even different arbors on the same neuron, will in general sample different aspects of that latent state; taken together, these complementary perspectives can tile the manifold of population activity without any single branch needing to carry a complete description. In this way, each neuron may act as a compressed “window” onto the brain's latent activity, analogous to how a small portion of a hologram still contains a recognizable image of the entire scene (in contrast to the strictly local information carried by a pixel). The reciprocal nature of this relationship is central. The aspects of the global state that are accessible to a neuron depend on its dendritic resonances and input sampling; conversely, the large-scale network can bias access to particular state dimensions by modulating the timing and frequency content of its projections. Through synaptic plasticity and activity-dependent tuning of intrinsic conductances, these two levels can become aligned over time, enabling local computations to remain dynamically coupled to the most relevant dimensions of the global cortical state. Crucially, invoking low-dimensional latent dynamics and the Johnson–Lindenstrauss lemma is not meant to render dendritic structure incidental. Access to latent structure is only the first step. The geometry, resonance gradients, and nonlinear mechanisms of dendrites determine how different components of that latent state are weighted, gated, combined, or suppressed, and thus shape the neuron's computational role. Two neurons may each receive projections of the same underlying population dynamics, yet their intrinsic structure determines which interactions are amplified, which are vetoed, and which nonlinear events are engaged. Dendrites therefore do not merely sample latent dynamics; they impose structure on how those dynamics are interpreted and transmitted, linking global population activity to local computation in a scale-consistent way.

Box 1 **The Johnson–Lindenstrauss lemma**

The Johnson–Lindenstrauss (JL) lemma (Johnson and Lindenstrauss, 1984) is a fundamental result in high-dimensional geometry that formalizes the intuition that the structure of a set of points can be preserved under projection into a much lower-dimensional space.

5.3.1 **Statement (informal)**

Given a set of *n* points (*x*_1_, ..., *x*_*n*_) in a high-dimensional space, there exists a linear mapping into a space of dimension

$$m=O\left(\frac{\log n}{\varepsilon^{2}}\right)$$

such that the pairwise distances between points are preserved to within a factor of (1±ε):

$$\left(1-\varepsilon \right)∥x_{i}-x_{j}{∥}^{2}\leq ∥f\left(x_{i}\right)-f\left(x_{j}\right){∥}^{2}\leq \left(1+\varepsilon \right)∥x_{i}-x_{j}{∥}^{2},$$

for all *i, j*.

5.3.2 **Intuition**

Even in a very high-dimensional space, a set of points with limited complexity (finite *n*) can be faithfully embedded into a much lower-dimensional space without significant distortion of geometry. The projection can be chosen at random, and with high probability it will satisfy the distance-preservation property.

5.3.3 **Relevance to neuroscience**

If large-scale neural population activity is well described by a low-dimensional latent trajectory, then the synaptic drive arriving at a dendritic branch can be viewed as a biased projection of that trajectory. Even a partial projection can preserve meaningful structure when the underlying dynamics are relatively low-dimensional. In neurons, the form of this projection is shaped by two factors: (i) the diversity of presynaptic partners converging onto the branch and (ii) the branch's frequency-selective resonance profile, which emphasizes particular temporal components of the global population state. Dendritic integration mechanisms further transform these inputs, determining which dimensions of the latent trajectory ultimately influence output. In this way, the spatial and temporal properties of resonant dendrites support both selective access to, and structured transformation of, global brain dynamics.

## 6 Implications and future directions

### 6.1 Multiscale recording approaches

Testing the resonant hierarchy framework will require measurement strategies that can bridge spatial scales, from dendritic compartments to whole-brain networks. Simultaneous intracranial population recordings and noninvasive measures such as scalp EEG or MEG offer a powerful means of linking local field potentials, spiking activity, and global oscillatory patterns within the same behavioral context. By examining how microscale events, such as bursts of gamma in a specific cortical column, align with or diverge from macroscale rhythms, these approaches can directly assess the degree of cross-scale alignment predicted by the framework.

Cortical layer-resolved recordings can further test how resonance properties vary with the source of input (e.g., feedforward vs. feedback) and with task context. Such measurements could test the hypothesis that frequency preferences observed at the laminar level are rooted in the resonance profiles of nearby dendrites, and that these preferences shift in predictable ways as behavioral demands change. Coupling laminar recordings with causal manipulations such as optogenetic or electrical stimulation tuned to specific frequencies could probe the directionality and frequency selectivity of inter-areal communication channels.

At the finest scale, intracellular patch-clamp recordings or subcellular imaging techniques (e.g., voltage-sensitive dye or genetically encoded voltage indicators) could be used to measure resonance directly at the synaptic and dendritic level. These experiments would enable precise quantification of how frequency tuning varies along the dendritic arbor, how it is modulated by neuromodulatory state, and how it adapts with experience or plasticity-inducing protocols. Such data could provide critical ground truth for connecting cellular-level mechanisms to the mesoscale and macroscale oscillatory phenomena observed *in vivo*.

By integrating these approaches, future work can move beyond correlational descriptions to causal, mechanistic tests of how oscillatory coordination emerges from, and shapes, the multiscale architecture of the brain.

### 6.2 Modeling latent dynamics across scales

A key test of the resonant hierarchy framework is whether it can account for neural population activity more parsimoniously or accurately than models that ignore cross-scale structure. Recent advances in machine learning provide new tools for this purpose, particularly deep generative models such as variational auto-encoders (VAEs) and related latent-variable approaches. When applied to multiscale data, such as simultaneously recorded intracranial population spiking and scalp EEG, these methods could infer a set of latent neural trajectories that jointly explain activity across recording modalities and spatial resolutions. A particularly influential example is Latent Factor Analysis via Dynamical Systems (LFADS) (Pandarinath et al., 2018), which uses recurrent neural networks to infer smooth latent dynamics from population spiking. Although LFADS has been primarily applied within single-scale datasets, its architecture and inference strategy could be adapted to test whether incorporating cross-scale constraints yields more accurate or interpretable latent representations.

Moving beyond conventional analyses of field potentials, these approaches can reveal whether distinct oscillatory components observed at different scales reflect projections of a shared low-dimensional latent process. For example, a latent variable evolving on a particular manifold might manifest as gamma-band fluctuations in local microcircuits, beta-band coherence between nearby cortical areas, and alpha-band coordination between distant networks, all reflecting a single underlying dynamical mode constrained by the system's resonant hierarchy.

To evaluate the plausibility of the framework, resonant hierarchy models should be quantitatively compared to simpler alternatives that lack explicit cross-scale structure. Model comparison metrics, such as predictive log-likelihood, explained variance, or out-of-sample decoding accuracy, can test whether incorporating scale-specific resonance improves predictions of neural activity and behavior. In addition, perturbation-in-the-model experiments, such as selectively altering the resonance properties of a particular scale, can generate falsifiable predictions for empirical validation.

By integrating multiscale recordings with latent dynamical modeling, future work can move toward a unified, computationally grounded account of how oscillatory coordination across the brain's structural hierarchy supports flexible cognition.

### 6.3 Stimulation as latent-state perturbation

In the resonant hierarchy framework, external stimulation is best understood not as a means of imposing fixed activity patterns, but as a way of biasing the brain's intrinsic latent dynamics toward particular regions of its state space. Transcranial electrical stimulation (tES), transcranial magnetic stimulation (TMS), deep brain stimulation (DBS), and other neuromodulatory approaches interact with ongoing network activity rather than replacing it; their effects depend on the phase, amplitude, and spectral content of the endogenous oscillations they encounter.

Recasting stimulation in this way shifts the experimental goal from matching a stimulation waveform to a hypothesized “optimal” frequency, to targeting the resonant scaffolds that constrain and shape multiscale coordination. By aligning stimulation parameters with these scaffolds (whether defined by dendritic resonance, laminar connectivity, or large-scale conduction delays) it may be possible to preferentially influence the oscillatory modes that mediate a desired computation. From this standpoint, DBS can be viewed as targeting resonant motifs in deep nuclei and their cortical partners, with clinical efficacy arising not simply from local suppression or excitation but from restoring or reshaping multiscale coordination regimes that have become pathologically disordered (e.g., Thevathasan et al., 2020).

Intracranial recordings during stimulation provide a direct window into these interactions, allowing researchers to characterize how applied fields modulate the geometry of latent neural trajectories. For example, closed-loop tES timed to the phase of a target oscillation can selectively amplify or suppress specific dimensions of neural state space, producing effects that propagate from the cellular to the network level in a scale-consistent manner. Such observations can refine biophysical models of how stimulation perturbs population activity, helping to disentangle the relative contributions of direct neuronal polarization, synaptic modulation, and network resonance.

Ultimately, precision neuromodulation may emerge not from stimulating specific neurons or anatomical loci, but from targeting the structural and dynamical features that anchor the brain's resonant hierarchy. By doing so, stimulation can nudge the system into functional regimes that support desired behaviors or cognitive states, offering a principled route toward individualized, state-dependent interventions.

### 6.4 Testable predictions and clinical implications

The resonant hierarchy framework generates a set of concrete, falsifiable predictions that can guide both basic and translational research. First, manipulations that alter dendritic resonance properties should systematically shift the spectral profile of population activity. For example, pharmacological modulation of *I*_*h*_ currents, *I*_*M*_ channels, or other conductances with known frequency-selective effects is predicted to bias the dominant oscillatory modes expressed locally and in connected networks. Such manipulations could be applied *in vitro, in vivo*, or in computational models to test the causal link between microscale resonance and macroscale coordination.

Second, disruptions to the structural substrates of the hierarchy are expected to impair the nesting and alignment of oscillations across scales. Neurodegenerative diseases, demyelination, or developmental disorders that alter axonal conduction delays may shift the optimal frequency bands for inter-areal communication, degrading temporal coordination. Similarly, pathologies affecting dendritic arborization, such as reduced apical branching in schizophrenia, may constrain the range of resonant modes available for local computation, producing measurable alterations in cross-frequency coupling or laminar-specific coherence (e.g., Glantz and Lewis, 2000; Rosoklija et al., 2000).

Third, this framework suggests novel diagnostic and therapeutic avenues for disorders characterized by dyscoordination of brain rhythms, including schizophrenia, autism spectrum disorder, and epilepsy (e.g., Uhlhaas and Singer, 2010; Simon and Wallace, 2016; Jiruska et al., 2013). This view aligns with the broader notion of “oscillopathy” articulated by Llinás et al. (1999), in which disrupted rhythms are treated as a core substrate of diverse neurological and psychiatric syndromes.

Because the resonant hierarchy links cellular, circuit, and network dynamics, oscillatory biomarkers can be interpreted in mechanistic terms, informing interventions targeted at the level most likely to restore functional alignment. For example, neuromodulatory treatments (whether pharmacological, electrical, or sensory) could be tuned to preferentially engage resonant modes that are underexpressed or to suppress pathological modes that dominate. In epilepsy, where hypersynchrony often emerges within a restricted frequency range (Jiruska et al., 2013; Jefferys et al., 2012), selectively dampening the corresponding resonant scaffold may prove more effective than broad-spectrum suppression.

By grounding predictions in biophysical and anatomical constraints, the resonant hierarchy framework offers a roadmap for empirical validation and a principled basis for clinical translation. Its multiscale scope allows hypotheses to be tested at the molecular, synaptic, circuit, and systems levels, enabling convergent evidence to accumulate across methods and species.

## 7 Conclusion

**Figure 5:**
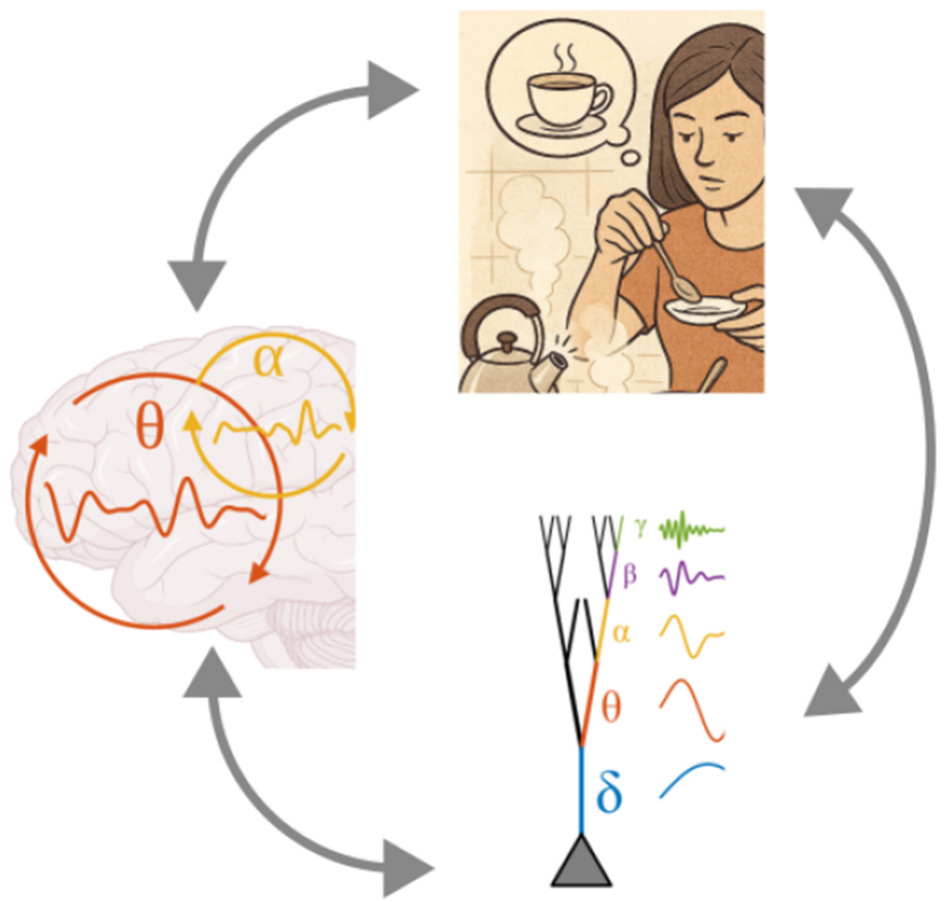
In the resonant hierarchy framework, neural oscillations unite hierarchies across all levels of brain function, from the hierarchy intrinsic to cognitive computations, to large-scale networks spanning the brain, to microscale structure of individual neurons.

The framework of resonant hierarchies advanced here proposes that oscillatory coordination in the brain is organized along nested structural scales, from the compartmental resonance of dendrites to the temporal regimes that shape inter-areal communication (Figure 5). In this view, canonical frequency bands are not tied to fixed cognitive functions, but instead define coordination regimes whose participation in behavior depends on anatomical placement, conduction constraints, and the resonance properties of the underlying microcircuitry. This scale-bridging organization is not incidental: frequency preferences originating at the level of ion channel distributions and dendritic morphology can propagate upward to influence the temporal structure of columns, cortical areas, and distributed networks, while global oscillatory states feed back to tune and reshape the resonance properties of their cellular substrates.

By shifting emphasis from one-to-one mappings between rhythms and cognitive “modules” toward a dynamical systems perspective grounded in biophysics and anatomy, the resonant hierarchy framework unites previously disparate observations under a common set of principles. It accommodates the diversity of oscillatory functions reported in the literature (e.g., apparent multiple roles of alpha activity) by recasting them as instances of shared temporal coordination regimes expressed in different structural contexts. This perspective also naturally generates testable predictions, linking changes in dendritic conductances, arborization, or axonal conduction to measurable shifts in network dynamics.

Future work at the intersection of multiscale recording, generative modeling, and targeted stimulation will be essential to test these predictions and to refine the mapping between resonance properties and functional coordination. If validated, the resonant hierarchy framework offers a principled path toward interventions that restore or optimize coordination across scales, whether in neurological disease or in the service of enhancing cognitive performance. In embracing this multiscale view, we move toward an account of brain rhythms not as byproducts of neural activity, but as fundamental scaffolds upon which the computational architecture of the nervous system is built.

## Data Availability

The original contributions presented in the study are included in the article/supplementary material, further inquiries can be directed to the corresponding author.
